# Raspberry Extract as a Strategy to Improve the Oxidative Stability of Pork Burgers Enriched with Omega-3 Fatty Acids

**DOI:** 10.3390/foods12081631

**Published:** 2023-04-13

**Authors:** Adrieni Santos de Oliveira, Bibiana Alves dos Santos, Carla Andressa Almeida Farias, Leticia Pereira Correa, Madison Willy Silva Cordeiro, Mariana Basso Pinton, Milene Teixeira Barcia, Roger Wagner, Alexandre José Cichoski, Juliano Smanioto Barin, José Manuel Lorenzo, Gema Nieto, Paulo Cezar Bastianello Campagnol

**Affiliations:** 1 Departamento de Tecnologia e Ciência dos Alimentos, Universidade Federal de Santa Maria, Santa Maria 97105-900, Rio Grande do Sul, Brazil; santosadrieni@gmail.com (A.S.d.O.); bialvesantos@gmail.com (B.A.d.S.); carlaaafarias@gmail.com (C.A.A.F.); pereiracorreal@gmail.com (L.P.C.); madison.cordeiro@ifmt.edu.br (M.W.S.C.); mbpinton@gmail.com (M.B.P.); milene.barcia@ufsm.br (M.T.B.); rogerwag@gmail.com (R.W.); cijoale@gmail.com (A.J.C.); juliano@ufsm.br (J.S.B.); 2 Centro Tecnológico de la Carne de Galicia, Adva. Galicia n° 4, Parque Tecnológico de Galicia, 32900 San Cibrao das Viñas, Spain; jmlorenzo@ceteca.net; 3 Área de Tecnología de los Alimentos, Facultad de Ciencias de Ourense, Universidade de Vigo, 32004 Ourense, Spain; 4 Department of Food Technology, Food Science and Nutrition, Faculty of Veterinary Sciences, Regional Campus of International Excellence “Campus Mare Nostrum”, University of Murcia, 30071 Murcia, Spain; gnieto@um.es

**Keywords:** lipid oxidation, linseed oil, pea protein, microwave hydrodiffusion and gravity, green chemistry

## Abstract

Hydrogelled emulsions (HEs) of linseed oil and pea protein (PP) were produced with four levels (0, 5, 7.5, and 10%) of raspberry extract obtained by a green extraction technique (microwave hydrodiffusion and gravity—MHG). HEs were applied in burgers to replace 50% of pork backfat content. The products’ technological, nutritional, oxidative, microbiological, and sensory properties were evaluated. Besides reducing the fat level by approximately 43%, the reformulation reduced the n-6/n-3 PUFA ratio to healthy levels, decreased the diameter reduction by 30%, and increased the cooking yield by 11%. Including 7.5 and 10% of raspberry extract in the HEs decreased the oxidative defects caused by the enrichment of the burgers with omega-3 fatty acids. In addition, the raspberry extract did not cause alterations in the mesophilic aerobic count and the burgers’ sensory profile.

## 1. Introduction

The high consumption of saturated fatty acids (SFA), which constitute over 40% of the lipid fraction in burgers, has been linked to negative health consequences [1]. Moreover, an imbalanced omega-6 to omega-3 ratio (between 15 to 20:1) in these products can exacerbate health risks, such as chronic diseases and certain types of cancer [2,3]. To address these concerns, incorporating oils rich in omega-3 fatty acids can improve the lipid profile of burgers. However, these healthful oils are susceptible to lipid oxidation, necessitating strategies to enhance their oxidative stability when applied to burgers.

Hydrogelation, a restructuring technique, is employed to modify oil consistency and mitigate the issues associated with its application in meat products [4]. Besides being cost-effective, it produces gels with appearance and functionality akin to animal fat. In a previous study, our research group utilized this technique to create hydrogelled emulsions (HEs) of linseed oil containing varying levels (0 to 20%) of pea protein (PP) as fat replacers in burgers. HEs with 10% PP significantly enhanced the burgers’ nutritional, technological, and sensory properties, but hydrogelation alone was insufficient to protect linseed oil from oxidative reactions fully [5]. Thus, further research is needed to explore strategies to bolster the oxidative stability of omega-3-rich HEs for industrial applications. One such approach involves incorporating bioactive compounds from natural sources into HEs [6,7].

The potential health benefits of functional foods enriched with bioactive compounds have garnered considerable attention in recent years. These bioactive compounds improve the nutritional profile of foods and offer protective effects against various diseases. Consequently, there is a growing interest in identifying and incorporating natural antioxidants into food formulations to enhance both the quality and shelf-life of products while promoting health benefits for consumers.

Raspberries (*Rubus ideaus* L.), a red fruit abundant in bioactive compounds, such as phenolics, organic acids, vitamins, minerals, and anthocyanins with potent antioxidant properties [8], can potentially enhance the oxidative stability of omega-3-enriched meat products. Previous studies have demonstrated that raspberry extracts can enhance the oxidative stability of fish oil (rich in n-3 PUFAs) [9] and various meat products, such as pastirma [10], meatballs [11], and burgers [12]. The mechanism by which raspberry extracts exert their antioxidant effects can be attributed to the presence of anthocyanins. Anthocyanins are natural pigments that possess strong antioxidant properties. They work by scavenging free radicals and preventing lipid oxidation, which can be detrimental to the quality and shelf life of meat products.

However, the extraction technique for obtaining bioactive compounds is a crucial factor. Employing environmentally harmful extraction methods or those leaving toxic residues contradicts the goal of improving food quality. Thus, eco-friendly extraction techniques are necessary. Farias et al. [13] demonstrated that the MHG technique effectively extracted many anthocyanins from raspberries using only water as a solvent and with low energy consumption, aligning with green chemistry principles.

Considering this context, the current study seeks to determine if raspberry extract, obtained using the eco-friendly MHG method, can improve the oxidative stability of burgers enriched with omega-3 fatty acids. To pursue this objective, the raspberry extract was combined with linseed oil. This blend was subsequently hydrogelled with pea protein (PP) and utilized as a fat substitute in burgers. In addition to evaluating the oxidative stability, the effects of this lipid reformulation on the burgers’ technological, nutritional, microbiological, and sensory characteristics were also evaluated.

## 2. Materials and Methods

### 2.1. Elaboration of the Raspberry Extract by MHG

Raspberry anthocyanin extracts were produced in a microwave oven (NEOS-GR, Milestone, Bergamo, Italy). Two hundred grams of frozen fruit were cut in half and submitted to microwave heating (600 Watts) for 9 min. These conditions were optimized previously by Farias et al. [13] to extract the highest concentration of bioactive constituents of raspberry. The raspberry extract was produced in triplicate.

### 2.2. Elaboration of Hydrogelled Emulsions Enriched with Raspberry Extract and Application in Burgers

The formulations of the hydrogelled emulsions (HEs) are shown in Table 1. The HEs were prepared according to the procedure described in de Lima Guterrez [5], and the raspberry extract was added to the aqueous phase. The burgers of the Control treatment were prepared with the following formulation: 78.4% pork, 20% pork backfat, 1.5% salt, and 0.1% garlic powder. The burgers of the treatments GE0, GE0.5, GE0.75, and GE1 were prepared with a 50% replacement of the pork backfat content of the Control by the HEs produced with 0 (HE0), 5 (HE5), 7.5 (HE7. 5) and 10% (HE10) of raspberry extract, respectively. Thus, the levels of raspberry extract in the burgers of treatments GE0, GE0.5, GE0.75, and GE1 were 0, 0.5, 0.75, and 1%, respectively. The burgers (100 g, 11 cm in diameter and 2.5 cm thick) were produced as described by de Lima Guterres et al. [5] and individually packaged in high-density polyethylene bags measuring 18 × 14 cm (Extrusa-Pack, São Paulo, SP, Brazil). The packages (50 µm thick) had oxygen and water vapor transmission rates of 1434 cm^3^/m^2^.day and 0.6 g/m^2^.day, respectively. The burgers were placed in styrofoam trays and kept in darkness for 12 days at 4 ± 0.1 °C. Three independent replicates of each HE and each batch of burgers were produced using the same ingredients and raw materials to limit experimental errors. Hence, 500 g of each HE and 30 burgers of each treatment were prepared in each independent replicate.

### 2.3. Determination of Chemical Composition and Fatty Acid Profile

The chemical composition (moisture, protein, lipids, and ash) and fatty acid profile of the burgers were analyzed in triplicate immediately after production. AOAC methods 950.46, 992.15, and 920.153 [14] were employed to determine moisture, protein, and ash contents, respectively. Lipid content was extracted and quantified using the Bligh and Dyer [15] method. Lipids were transesterified to fatty acid methyl esters (FAMEs) using the Hartman and Lago [16] method. FAMEs were measured according to the conditions outlined by de Lima Guterres et al. [5], with results expressed as grams of fatty acids per 100 g of the product. Atherogenicity (*AI*) and thrombogenicity (*TI*) indices were computed using the following equations [17]:

(1) $$AI=\frac{C12:0+\left(4\times C14:0\right)+C16:0}{\left(ƩPUFA\right)+\left(ƩMUFA\right)}$$

(2) $$TI=\frac{C14:0+C16:0+C18:0}{\left(0.5\times ƩMUFA\right)+\left(0.5\times n-6\right)+\left(3\times n-3\right)+(\frac{n-3}{n-6})}$$

### 2.4. Technological Properties

The weight and the diameter of three raw burgers per treatment from each independent replicate were measured. Next, the burgers were cooked to 72 °C in the core. Cooking was performed on a grill (Multi Grill, Britânia, Brazil) set at 150 °C. The burgers were turned every 2 min to ensure even cooking. The cooked burgers were cooled to 25 °C, and their weight and diameter were again measured. The following equations were used to calculate the *diameter reduction* and the *cooking yield*:

(3) $$\%diameterreduction=\frac{{diameter}_{raw}-{diameter}_{cooked}}{{diameter}_{raw}}\times 100$$

(4) $$\%cookingyield=\frac{{weight}_{cooked}}{{weight}_{raw}}\times 100$$

### 2.5. Determination of Instrumental Color

A colorimeter (Model CR-400, Konica Minolta Sensing Inc., Osaka, Japan) was used to measure the burgers’ L*, a*, and b* values during the storage (1, 4, 8, and 12 days of storage). The equipment operated with the following parameters: illuminant D65, observation angle of 10°, and aperture of 1.5 cm. The colorimeter was calibrated before each measurement session using a standard white plate. Two raw burgers per treatment from each independent replicate were used for instrumental color determination on each analysis day. The plastic packaging was previously removed, and the color determination was performed at six different points of each burger. ΔE values were calculated for each treatment by comparing the L*, a*, and b* values from days 4, 8, and 12 with day 1 of storage according to the equation:

ΔE = [(L* − L*_0_)^2^ + (a* − a*_0_)^2^ + (b* − b*_0_)^2^]^0.5^ (5)

ΔE is calculated as the square root of the sum of squared differences between the L*, a*, and b* coordinates for a given treatment on days 4, 8, and 12 compared to day 1 [18].

### 2.6. Determination of pH and TBARS Values

The pH and TBARS values of the burgers were measured on days 1, 4, 8, and 12 of storage. Two raw burgers per treatment from each independent replicate were analyzed in triplicate on each day of storage. pH measurements were taken using a pre-calibrated pH meter (Model 130 MA; Mettler Toledo, São Paulo, Brazil) with pH 4.0 and 7.0 buffer solutions (Merck, Germany). The readings were obtained from a homogenized solution consisting of 5 g of sample and 50 mL of distilled water. TBARS values were expressed as mg of malondialdehyde per kg of the sample, and the analytical procedure was performed using the methodology described by Bruna et al. [19].

### 2.7. Microbiological Analysis

The quantification of aerobic mesophilic microorganisms was performed on days 1, 4, 8, and 12 of storage, according to Silva et al. [20]. Two raw burgers per treatment from each independent replicate were analyzed in triplicate on each day of storage.

### 2.8. Sensory Analysis

The sensory profile of the burgers was determined using a descriptive sensory analysis following the procedures outlined by Wang et al. [21]. The Federal University of Santa Maria’s Human Research Ethics Committee approved the protocol (CAAE 28514820.0.0000.5346).

Fifteen tasters, aged between 20 and 55 years, were selected using triangular and basic taste recognition tests according to Stone and Sidel’s [22] methodology. In a Free Choice Profiling session, the tasters defined descriptor terms for characterizing samples and evaluating color and aroma changes related to lipid oxidation during storage.

On day 1, a descriptive analysis was conducted to assess the impact of lipid reformulation on the sensory profile of cooked burgers, focusing on fishy aroma, characteristic aroma, fishy taste, characteristic taste, and softness. Burgers were cooked, cut into eight pieces, and kept at 60 °C in aluminum foil until the sensory analysis. Samples not evaluated within 60 min were discarded.

During storage (days 1, 4, 8, and 12), a descriptive analysis was performed to examine the effect of lipid oxidation on the color and aroma of raw burgers. Descriptors included characteristic color, oxidized color, characteristic aroma, and rancid aroma. Raw burgers were removed from packaging, placed in Petri dishes, and kept at 4 °C. Samples not analyzed within 30 min were discarded.

Sensory analysis was conducted in individual booths with fluorescent lighting. Samples were coded with three random numbers and presented to panelists in a monadic manner using a completely balanced block [23]. A 9 cm unstructured scale was used to evaluate the descriptors (1—little and 9—very).

### 2.9. Statistical Analysis

The entire experiment was conducted three times on separate days. Levene and Shapiro–Wilk tests confirmed that the data followed a normal distribution. Grubbs test was employed to remove outliers (*p* < 0.05). A generalized linear model was used to analyze the results. Fixed effects in the model included “treatments” and “storage time.” Repetitions and tasters were incorporated into the model as random effects. The interaction between “treatments” and “storage time” was also assessed. Tukey’s test was utilized for mean comparisons (*p* < 0.05).

## 3. Results and Discussion

### 3.1. Chemical Composition and Fatty Acid Profile

The chemical composition of the bioactive compound-enriched burgers is presented in Table 2. The moisture content in the reformulated samples was higher (*p* < 0.01) than the Control, which was expected due to the presence of 50–60% water in the HEs. The protein and ash contents were unaffected (*p* > 0.05) by substituting pork backfat with HEs. However, the reformulated samples had around 43% less fat than the Control (*p* < 0.001).

Apart from the reduced fat content, the reformulated burgers also exhibited a more nutritionally favorable fatty acid profile for human health (Table 3). The primary nutritional advantage of lipid reformulation was the 50% reduction in lauric (C12:0), myristic (C14:0), and palmitic acids (C16:0), which are positively associated with increased LDL cholesterol (Low-Density Lipoproteins) [2]. Moreover, stearic acid (C18:0) content was reduced by approximately 50% in the reformulated samples, which is beneficial for the product’s nutritional quality. Although stearic acid has a neutral impact on triglycerides and total cholesterol, its overall effect on cardiovascular disease development remains uncertain [24].

Increasing omega-3 fatty acids and decreasing omega-6 fatty acids also offer nutritional benefits to burgers, as consuming foods with an n-6/n-3 ratio greater than 4 is positively linked to inflammatory reactions and cardiovascular diseases [25]. The lipid reformulation’s nutritional advantages were further demonstrated by improvements in other critical nutritional indices, such as an increased PUFA/SFA ratio and decreased atherogenicity and thrombogenicity indices.

### 3.2. Technological Properties

**Figure 1:**
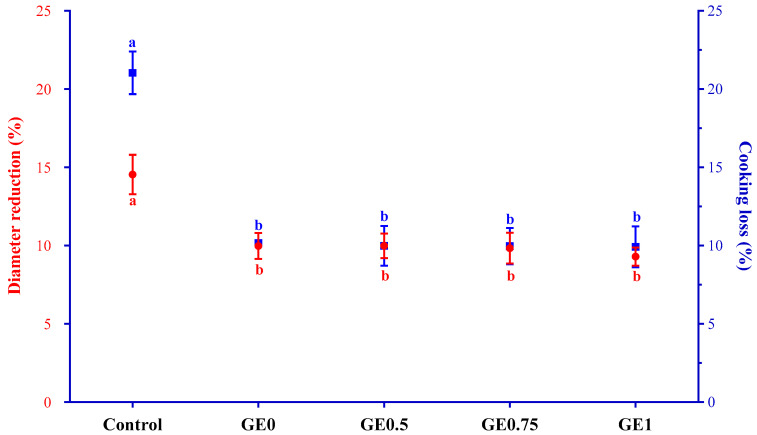
Technological properties of pork burgers enriched with bioactive compounds. Distinct letters demonstrated significant variations in Tukey’s analysis (*p* < 0.05). Error bars represent the standard error of the mean. Regimens: Control group: 20% pork back fat; GE0, GE0.5, GE0.75, and GE: 50% replacement of pork back fat using hydrogel emulsion derived from linseed oil and pea protein, incorporating 0, 0.5, 0.75, and 1% raspberry extract, correspondingly.

The diameter reduction and cooking yield results are shown in Figure 1. The reformulated burgers showed a diameter reduction after cooking approximately 30% less than the Control (*p* < 0.001). Furthermore, lipid reformulation increased the cooking yield by about 11% (*p* < 0.001). These results indicate that the reformulated products retained more water and fat during cooking, which benefits their sensory quality. This improvement in technological quality is due to the high water and fat retention capacity of the PP that was added to HE [26]. Agreeing with these results, de Lima Guterres et al. [5] also reported better burger cooking properties with the replacement of animal fat by HEs made with 10 to 20% PP. Other studies have also reported the beneficial effect of PP on the technological properties of restructured [27] and emulsified [28] meat products. The addition of raspberry extract did not change the analyzed cooking properties.

### 3.3. Instrumental Color

The L*, a*, and b* values of the burgers shortly after production (day 1) are presented in Table 4. There were no significant differences (*p* > 0.05) in the L*, a*, and b*values between the sample prepared with HE without raspberry extract (GE0) and the Control. This result aligns with a previous study that found no differences in L*, a*, and b* values when replacing animal fat with HEs made from linseed oil containing up to 10% PP [5]. Adding the raspberry extract to the HEs did not alter the values of the burgers’ L*, a*, and b*. This finding is significant because numerous studies have reported that incorporating natural extracts can negatively impact the color of meat products, rendering their use unfeasible [29,30,31].

**Figure 2:**
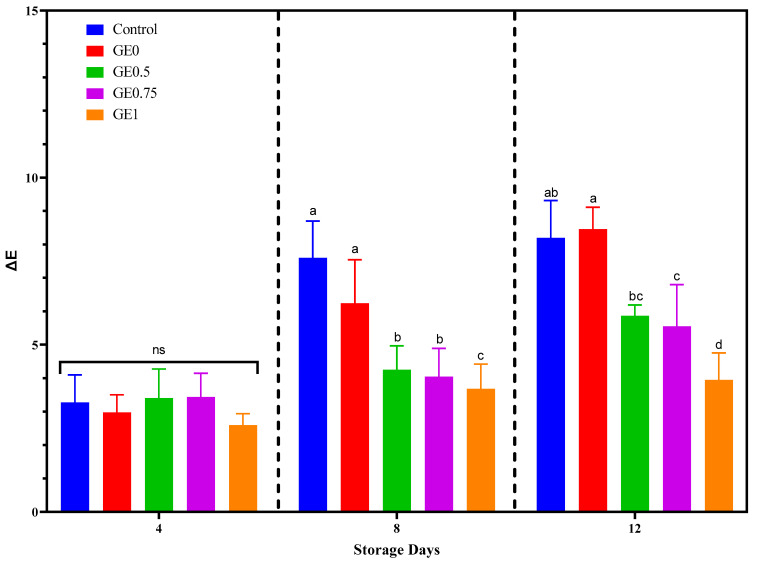
ΔE values of burgers calculated for each treatment by comparing the color differences on days 4, 8, and 12 with day 1. Distinct letters demonstrated significant variations in Tukey’s analysis (*p* < 0.05). Error bars represent the standard error of the mean. Regimens: Control group: 20% pork back fat; GE0, GE0.5, GE0.75, and GE: 50% replacement of pork back fat using hydrogel emulsion derived from linseed oil and pea protein, incorporating 0, 0.5, 0.75, and 1% raspberry extract, correspondingly.

ΔE values were calculated to display the color differences between treatments immediately after production (day 1) and after 4, 8, and 12 days of refrigerated storage (Figure 2). After four days of refrigerated storage, all treatments had ΔE values lower than 5, indicating that the L*, a*, and b* values remained close to their initial levels, and consumers would struggle to detect color differences [32,33]. After eight days of refrigerated storage, the Control and GE0 samples exhibited visually perceptible ΔE values (above 5). However, samples with raspberry extract (GE0.5, GE0.75, and GE1) maintained ΔE values below 5 during this period, with the GE1 sample staying beneath the visually detectable threshold until the end of storage (day 12).

### 3.4. pH and TBARS

**Figure 3:**
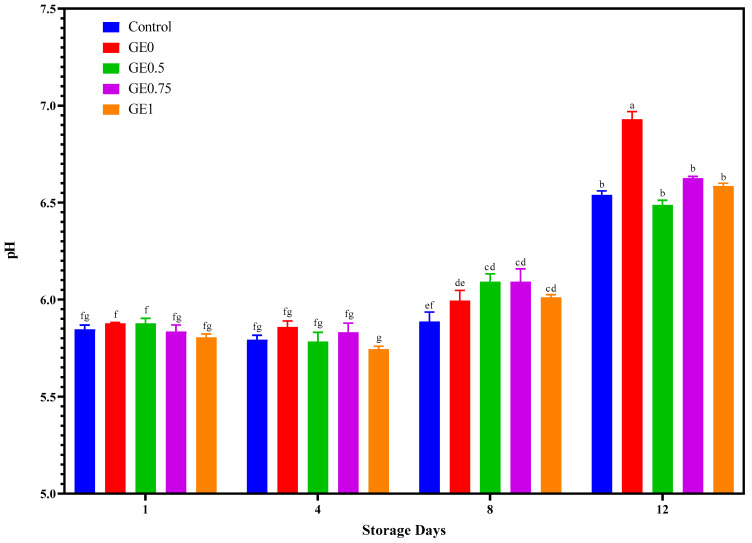
pH values of bioactive compound-enriched pork burgers. Distinct letters demonstrated significant variations in Tukey’s analysis (*p* < 0.05). Error bars represent the standard error of the mean. Regimens: Control group: 20% pork back fat; GE0, GE0.5, GE0.75, and GE: 50% replacement of pork back fat using hydrogel emulsion derived from linseed oil and pea protein, incorporating 0, 0.5, 0.75, and 1% raspberry extract, correspondingly.

The interaction between the fixed factors (treatments and storage time) significantly (*p* < 0.001) influenced pH evolution (Figure 3). Right after manufacturing, the pH values of all samples were between 5.8 and 5.9 (*p* > 0.05). These pH values are within the range commonly reported for burgers [34].

The pH values of all treatments remained stable until the 4th day of storage (*p* > 0.05). From the 8th day of storage, pH values increased, with the highest increase observed in the GE0 sample at the end of storage. This increase is due to proteolytic reactions that, when degrading proteins, release alkaline compounds [35]. De Lima Guterres et al. [5] also observed a more pronounced increase in pH during storage for burgers containing PP, which might be associated with proteolytic and oxidative reactions. In the present study, treatments with raspberry extract showed lower pH values after 12 days of storage than GE0 (*p* < 0.001). Therefore, this result suggests that the raspberry extract may have protected PP from oxidative reactions since the anthocyanins present in the extract are more stable at a lower pH range [10].

**Figure 4:**
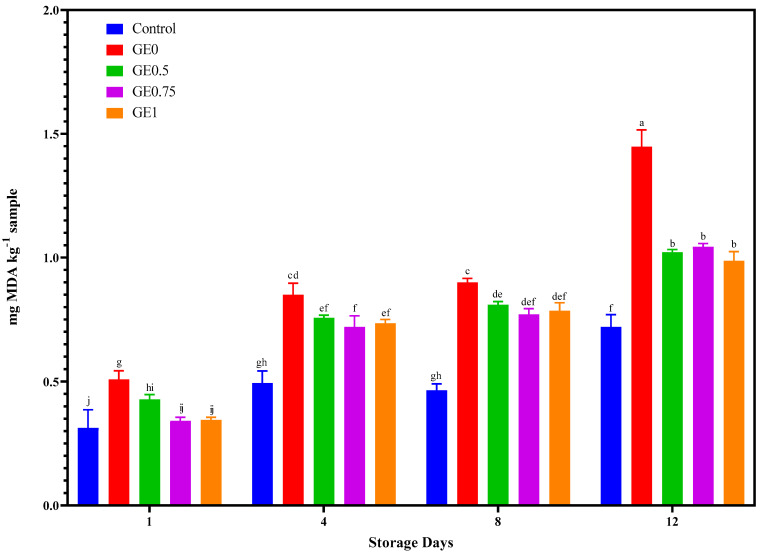
TBARS values for bioactive compound-enriched pork burgers. Distinct letters demonstrated significant variations in Tukey’s analysis (*p* < 0.05). Error bars represent the standard error of the mean. Regimens: Control group: 20% pork back fat; GE0, GE0.5, GE0.75, and GE: 50% replacement of pork back fat using hydrogel emulsion derived from linseed oil and pea protein, incorporating 0, 0.5, 0.75, and 1% raspberry extract, correspondingly.

TBARS values were significantly influenced (*p* < 0.001) by the interaction between treatments and storage time (Figure 4). At the beginning of storage, the TBARS values of the GE0 sample were already significantly higher than the Control. Throughout storage, the Control exhibited lower TBARS values than the reformulated treatments, except on day 1 when samples GE0.75 and GE1 had similar values. This difference can be easily explained by examining omega-3 fatty acid content differences between the Control and the reformulated samples (Table 2), as this fatty acid is highly susceptible to oxidative reactions [36]. This increase in lipid oxidation due to the enrichment with omega-3 fatty acids is commonly reported and represents one of the main challenges to overcome when incorporating healthy oils into meat products [37,38].

Samples containing raspberry extract (GE0.5, GE0.75, and GE1) demonstrated lower (*p* < 0.001) TBARS values than the GE0 sample (without raspberry extract) throughout storage. The most significant difference was observed at the end of storage (day 12) when samples GE0.5, GE0.75, and GE1 had TBARS values 30% lower than GE0. It should be noted that although the samples with raspberry extract exhibited greater lipid oxidation than the Control, their TBARS values remained below the sensorially detectable limit (<1.0 mg of MDA/kg of the sample) throughout storage [39].

Farias et al. [13] reported that the raspberry extract used in this study contained a high concentration of anthocyanins, with cyanidin-3-dihexoside being the major compound. This finding explains the antioxidant effect observed in samples GE0.5, GE0.75, and GE1, as anthocyanins have the ability to stabilize free radicals by donating hydrogen atoms. This process reduces the rate of oxidative reactions [30]. In agreement with these results, Aksu et al. [10] reported improved oxidative stability in pastirma supplemented with 4 and 5% aqueous raspberry extract.

### 3.5. Microbiological and Sensory Analysis

**Figure 5:**
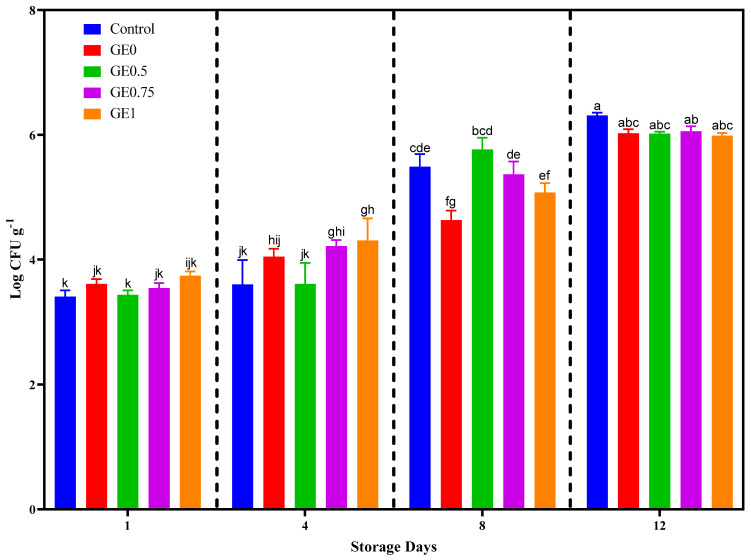
Mesophilic aerobic microorganisms count (Log CFU g^−1^) in bioactive compound-enriched pork burgers. Distinct letters demonstrated significant variations in Tukey’s analysis (*p* < 0.05). Error bars represent the standard error of the mean. Regimens: Control group: 20% pork back fat; GE0, GE0.5, GE0.75, and GE: 50% replacement of pork back fat using hydrogel emulsion derived from linseed oil and pea protein, incorporating 0, 0.5, 0.75, and 1% raspberry extract, correspondingly.

The interaction between “treatments” and “storage time” had a significant impact (*p* < 0.001) on the development of mesophilic aerobic microorganisms (Figure 5). In all treatments, the initial count of aerobic mesophilic microorganisms was below 4 log CFU g^−1^ (*p* > 0.05). After 12 days of storage, the count reached values signifying the end of the product’s shelf life (6 log CFU g^−1^) [40] in all treatments. The raspberry extract did not interfere with the evolution of the mesophilic aerobic count. Similarly, other studies did not report an antimicrobial effect of extracts with a high content of phenolic compounds in meat products [41,42,43].

**Figure 6:**
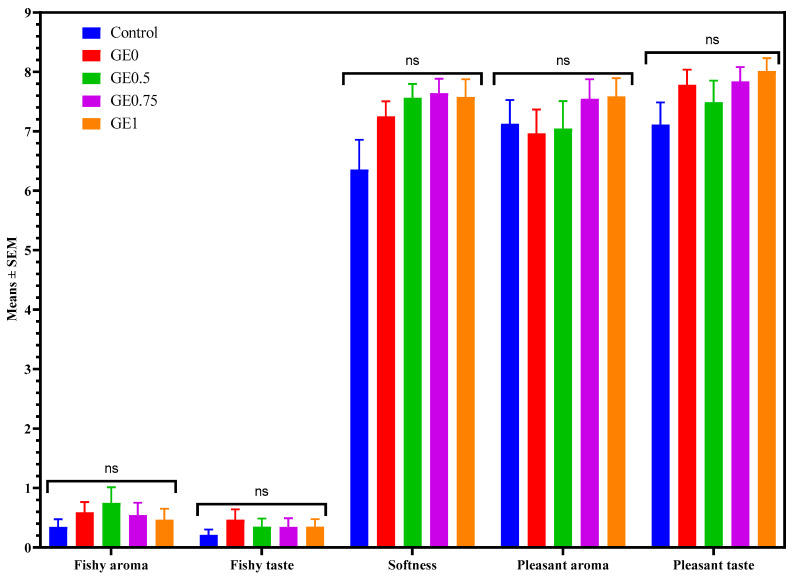
Results of the sensory evaluation performed shortly after manufacturing (day 1) of the burgers enriched with bioactive compounds. Distinct letters demonstrated significant variations in Tukey’s analysis (*p* < 0.05). Error bars represent the standard error of the mean. Regimens: Control group: 20% pork back fat; GE0, GE0.5, GE0.75, and GE: 50% replacement of pork back fat using hydrogel emulsion derived from linseed oil and pea protein, incorporating 0, 0.5, 0.75, and 1% raspberry extract, correspondingly. n.s. (not significant).

The results of the sensory evaluation performed shortly after manufacturing (day 1) of the burgers are shown in Figure 6. Previous studies reported that adding linseed oil to burgers gave a fishy aroma and taste, impairing the products’ sensory quality [7]. In this study, adding HE containing 25% linseed oil did not increase the sensory perception of the burgers’ fishy aroma and fishy taste (*p* > 0.05). Furthermore, replacing pork backfat with HE without raspberry extract did not significantly affect the attributes “softness”, “characteristic aroma”, and “characteristic taste” of the burgers. The enrichment of HE with raspberry extract also did not cause significant changes in the sensory descriptors. This result is essential considering that some studies have reported a decrease in the sensory quality of meat products supplemented with plant extracts [44,45].

**Figure 7:**
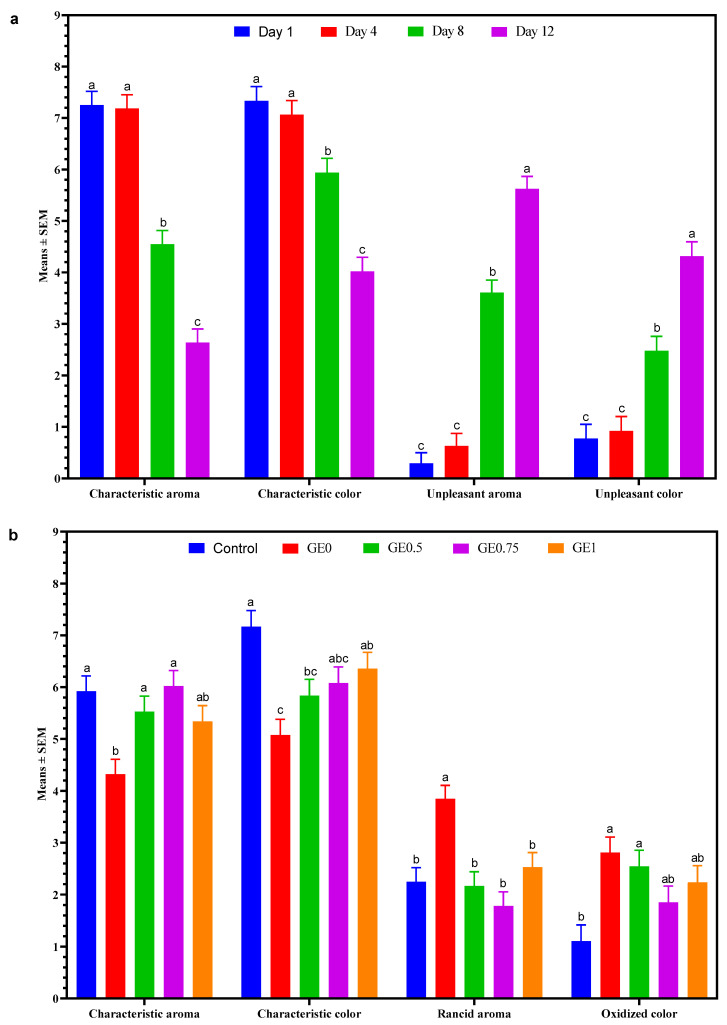
Sensory descriptors of color and aroma related to lipid oxidation analyzed during burger storage (1, 4, 8, and 12 days). (**a**,**b**): effects of the factors “storage time” and “treatments”, respectively. Distinct letters demonstrated significant variations in Tukey’s analysis (*p* < 0.05). Error bars represent the standard error of the mean. Regimens: Control group: 20% pork back fat; GE0, GE0.5, GE0.75, and GE: 50% replacement of pork back fat using hydrogel emulsion derived from linseed oil and pea protein, incorporating 0, 0.5, 0.75, and 1% raspberry extract, correspondingly.

In this study, changes in sensory descriptors of color and aroma related to lipid oxidation were also analyzed during burger storage (1, 4, 8, and 12 days) (Figure 7). No significant interactions were observed between the factors “storage time” and “treatments” in any sensory descriptor analyzed (*p* > 0.05). For this reason, the effects of the factors “storage time” and “treatments” are presented separately in Figure 7a and Figure 7b, respectively.

The sensory descriptors analyzed were not significantly affected until the 4th day of storage. Significant changes in oxidative stability were sensorially identified from the 8th day of storage, being even more potentiated at the end of storage (day 12) (*p* < 0.05). These results align with the evolution of ΔE (Figure 2) and TBARS (Figure 4) values.

The G0 sample showed a significant reduction during storage in the descriptors “characteristic aroma” and “characteristic color” notes concerning the Control. In addition, G0 had the highest score in the “rancid aroma” attribute among all treatments. This result is consistent with the higher TBARS values in G0 during storage (Figure 4). G0 also exhibited a higher score for the “oxidized color” attribute compared to the Control, which aligns with both the TBARS results and the progression of ΔE values (Figure 2). These findings, therefore, confirm that substituting pork backfat with HE-containing a high concentration of omega-3 fatty acids significantly compromised the oxidative quality of the burgers. Other researchers obtained similar results when applying oils rich in omega-3 fatty acids to meat products [46,47,48]. Incorporating raspberry extract in HE reduced the sensory defects observed in the analyzed descriptors. The best results were observed in samples containing HEs produced with 7.5% and 10% of raspberry extract (GE0.75 and GE1), corresponding to 0.75% and 1% of raspberry extract in the burger, respectively. The fact that samples GE0.75 and GE1 presented scores significantly similar to the Control in all sensory descriptors analyzed during storage is well correlated with the evolution of ΔE (Figure 2) and TBARS (Figure 4) values. Therefore, these results prove that the raspberry extract reduced the lipid oxidation caused during storage by the enrichment of burgers with omega-3 fatty acids.

## 4. Conclusions

In this study, substituting 50% of pork backfat with HEs created from linseed oil and PP led to improved nutritional and technological qualities in the burgers without impacting the growth of mesophilic aerobic microorganisms. However, both instrumental and sensory analyses revealed a decrease in the burgers’ oxidative stability due to the proposed lipid reformulation. Including 7.5% and 10% raspberry extract in the HEs mitigated the oxidative issues resulting from omega-3 fatty acid enrichment, rendering them undetectable to the senses. Consequently, the study’s findings demonstrate the feasibility of producing healthier burgers with satisfactory technological, oxidative, and sensory qualities by replacing 50% animal fat with HEs composed of linseed oil, PP, and raspberry extract.

## Figures and Tables

**Table 1:** Formulations of hydrogelled emulsions of linseed oil and pea protein enriched with raspberry extract.

(%)	HE0	HE5	HE7.5	HE10
Linseed oil	25	25	25	25
Pea protein	10	10	10	10
Tween 80	1	1	1	1
Carrageenan	4	4	4	4
Raspberry extract	0	5	7.5	10
Water	60	55	52.5	50
Total	100	100	100	100

**Table 2:** Chemical composition (%) of pork burgers enriched with bioactive compounds.

	Control	GE0	GE0.5	GE0.75	GE1	SEM	Sig.
Moisture	65.1 ^b^	67.1 ^a^	66.9 ^a^	66.6 ^a^	67.0 ^a^	1.4	**
Protein	19.4 ^a^	18.3 ^a^	18.7 ^a^	18.4 ^a^	20.0 ^a^	0.4	n.s.
Lipid	21.8 ^a^	12.3 ^b^	12.5 ^b^	12.6 ^b^	12.3 ^b^	0.4	***
Ash	2.9 ^a^	2.7 ^a^	3.1 ^a^	2.4 ^a^	2.5 ^a^	0.2	n.s.

Mean values in the same row with identical letters displayed no significant variation (*p* > 0.05), according to Tukey’s analysis. Groupings: Control group: 20% pork back fat; GE0, GE0.5, GE0.75, and GE: 50% replacement of pork back fat with hydrogel emulsion made from linseed oil and pea protein, containing 0, 0.5, 0.75, and 1% raspberry extract, respectively. SEM: Standard error of the mean. Sig: n.s. (not significant), ** (*p* < 0.01), *** (*p* < 0.001).

**Table 3:** Fatty acid composition (represented as grams of fatty acids per 100 g of the sample) in pork burgers enhanced with bioactive components.

	Control	GE0	GE0.5	GE0.75	GE1	SEM	Sig
**C12:0**	0.02 ^a^	0.01 ^b^	0.009 ^b^	0.009 ^b^	0.008 ^b^	0.00	***
**C14:0**	0.34 ^a^	0.14 ^c^	0.17 ^b^	0.16 ^bc^	0.15 ^c^	0.01	***
**C14:1**	0.012 ^a^	0.007 ^b^	0.006 ^b^	0.008 ^b^	0.007 ^b^	0.00	***
**C15:0**	0.034 ^a^	0.017 ^b^	0.017 ^bc^	0.018 ^b^	0.013 ^c^	0.00	***
**C16:0**	4.76 ^a^	2.35 ^c^	2.52 ^b^	2.49 ^b^	2.45 ^bc^	0.10	***
**C16:1**	0.64 ^a^	0.27 ^bc^	0.30 ^b^	0.29 ^bc^	0.24 ^c^	0.02	***
**C17:0**	0.14 ^a^	0.07 ^b^	0.07 ^b^	0.07 ^b^	0.07 ^b^	0.00	***
**C:17:1 n10 cis**	0.09 ^a^	0.04 ^b^	0.04 ^b^	0.04 ^b^	0.04 ^b^	0.00	***
**C18:0**	2.85 ^a^	1.45 ^c^	1.59 ^b^	1.59 ^b^	1.36 ^c^	0.06	***
**C18:1 n9 cis**	8.51 ^a^	4.34 ^c^	4.51 ^b^	4.54 ^b^	4.36 ^c^	0.17	***
**C18:2 n6 trans**	0.04 ^a^	0.02 ^b^	0.02 ^b^	0.02 ^b^	0.02 ^b^	0.00	***
**C18:2 n6 cis**	3.58 ^a^	2.12 ^b^	1.96 ^c^	2.07 ^bc^	2.01 ^bc^	0.06	***
**C20:0**	0.04 ^a^	0.02 ^b^	0.02 ^b^	0.02 ^b^	0.02 ^b^	0.00	***
**C18:3 n6**	0.008 ^a^	0.005 ^b^	0.003 ^c^	0.007 ^ab^	0.006 ^ab^	0.00	***
**C18:3 n3**	0.38 ^c^	1.25 ^ab^	1.09 ^b^	1.07 ^b^	1.40 ^a^	0.04	***
**C20:2 n6**	0.17 ^a^	0.07 ^b^	0.07 ^b^	0.08 ^b^	0.06 ^b^	0.00	***
**C22:0**	0.009 ^bc^	0.012 ^a^	0.008 ^c^	0.011 ^ab^	0.009 ^bc^	0.00	**
**C20:3 n3**	0.025 ^a^	0.013 ^bc^	0.009 ^d^	0.016 ^b^	0.009 ^cd^	0.00	***
**C20:3 n6**	0.027 ^a^	0.014 ^b^	0.014 ^b^	0.015 ^b^	0.012 ^b^	0.00	***
**C20:4 n6**	0.13 ^a^	0.07 ^b^	0.05 ^d^	0.07 ^bc^	0.06 ^cd^	0.00	***
**C24:0**	0.002 ^c^	0.004 ^b^	0.004 ^b^	0.004 ^ab^	0.005 ^a^	0.00	***
**C24:1 n9**	0.002 ^a^	0.001 ^b^	0.001 ^c^	0.001 ^c^	0.000 ^d^	0.00	***
**C22:6 (DHA)**	0.008 ^a^	0.004 ^b^	0.003 ^b^	0.004 ^b^	0.004 ^b^	0.00	***
**∑SFA**	8.61 ^a^	4.27 ^c^	4.60 ^b^	4.57 ^b^	4.23 ^c^	0.17	***
**∑MUFA**	13.35 ^a^	8.10 ^b^	7.99 ^b^	8.09 ^b^	8.12 ^b^	0.22	***
**∑PUFA**	4.33 ^a^	3.55 ^b^	3.22 ^c^	3.33 ^bc^	3.56 ^b^	0.04	***
**PUFA/SFA**	0.32 ^c^	0.44 ^a^	0.40 ^b^	0.41 ^b^	0.44 ^a^	0.00	***
**∑n-3**	0.44 ^c^	1.29 ^ab^	1.12 ^b^	1.11 ^b^	1.43 ^a^	0.04	***
**∑n-6**	3.99 ^a^	2.32 ^b^	2.14 ^c^	2.28 ^bc^	2.19 ^bc^	0.07	***
**n-6/n-3**	9.10 ^a^	1.80 ^bc^	1.91 ^b^	2.06 ^b^	1.53 ^c^	0.30	***
**AI**	0.35 ^a^	0.25 ^c^	0.29 ^b^	0.28 ^bc^	0.26 ^bc^	0.00	***
**TI**	0.65 ^a^	0.39 ^c^	0.44 ^b^	0.43 ^b^	0.39 ^c^	0.01	***

Mean values in the same row with identical letters displayed no significant variation (*p* > 0.05), according to Tukey’s analysis. Groupings: Control group: 20% pork back fat; GE0, GE0.5, GE0.75, and GE: 50% replacement of pork back fat with hydrogel emulsion made from linseed oil and pea protein, containing 0, 0.5, 0.75, and 1% raspberry extract, respectively. SEM: Standard error of the mean. Sig: ** (*p* < 0.01), *** (*p* < 0.001). SFA = saturated fatty acids; MUFA = monounsaturated fatty acids; PUFA = polyunsaturated fatty acids; n-6 = omega-6; n-3 = omega-3. AI: atherogenic index; TI: thrombogenic index.

**Table 4:** Instrumental color (day 1) of pork burgers enriched with bioactive compounds.

	Control	GE0	GE0.5	GE0.75	GE1	SEM	Sig.
L*	56.6 ^a^	58.3 ^a^	55.0 ^a^	54.3 ^a^	51.2 ^a^	1.3	n.s.
a*	6.9 ^a^	7.1 ^a^	6.05 ^a^	6.5 ^a^	5.7 ^a^	0.6	n.s.
b*	16.2 ^a^	17.0 ^a^	16.8 ^a^	15.5 ^a^	16.2 ^a^	0.8	n.s.

Mean values in the same row with identical letters displayed no significant variation (*p* > 0.05), according to Tukey’s analysis. Groupings: Control group: 20% pork back fat; GE0, GE0.5, GE0.75, and GE: 50% replacement of pork back fat with hydrogel emulsion made from linseed oil and pea protein, containing 0, 0.5, 0.75, and 1% raspberry extract, respectively. SEM: Standard error of the mean. Sig: n.s. (not significant).

## Data Availability

The data presented in this study are available on request from the corresponding author.
